# The influence of cortisol co-secretion on clinical characteristics and postoperative outcomes in unilateral primary aldosteronism

**DOI:** 10.3389/fendo.2024.1369582

**Published:** 2024-04-30

**Authors:** Yiran Jiang, Lihua Zhou, Cui Zhang, Tingwei Su, Lei Jiang, Weiwei Zhou, Xu Zhong, Luming Wu, Weiqing Wang

**Affiliations:** ^1^ Shanghai Key Laboratory for Endocrine Tumors, Shanghai Clinical Centre for Endocrine and Metabolic Diseases, Ruijin Hospital, Shanghai Jiaotong University School of Medicine, Shanghai, China; ^2^ Laboratory for Endocrine and Metabolic diseases of Institute of Health Science, Shanghai Jiaotong University School of Medicine and Shanghai Institutes for Biological Sciences, Chinese Academy of Sciences, Shanghai, China

**Keywords:** primary aldosteronism, cortisol, complete clinical success, cosyntropin stimulation test, KCNJ5

## Abstract

**Context:**

The prevalence of unilateral primary aldosteronism (UPA) with cortisol co-secretion varies geographically.

**Objective:**

To investigate the prevalence and clinical characteristics of UPA with cortisol co-secretion in a Chinese population.

**Design:**

Retrospective cohort study.

**Methods:**

We recruited 580 patients with UPA who underwent cosyntropin stimulation test (CST) after the 1-mg dexamethasone suppression test (DST) and retrospectively analyzed the clinical characteristics and postoperative outcomes of UPA with and without cortisol co-secretion.

**Results:**

UPA with cortisol co-secretion (1 mg DST>1.8 ug/dL) was identified in 65 of 580 (11.2%) patients. These patients were characterized by older age, longer duration of hypertension, higher concentration of plasma aldosterone and midnight cortisol, lower adrenocorticotropic hormone (ACTH) and dehydroepiandrosterone sulfate (DHEAS), larger tumor diameter, and more history of diabetes mellitus. Cortisol and aldosterone levels were higher and DHEAS level was lower in UPA with cortisol co-secretion at 0–120 min after CST. Among 342 UPA patients with *KCNJ5* gene sequencing and follow-up results, the complete clinical success rate was lower in UPA with cortisol co-secretion (33.3% vs. 56.4%, P<0.05); the complete biochemical success rate and *KCNJ5* mutation did not differ between the two groups. Age, tumor size, and ACTH were independent predictors of UPA with cortisol co-secretion. Sex, BMI, duration of hypertension, *KCNJ5* mutation, and cortisol co-secretion were independent predictors for complete clinical success in UPA after surgery.

**Conclusions:**

UPA with cortisol co-secretion is not uncommon in China, but the clinical features were distinctly different from those without co-secretion. Cortisol co-secretion is an independent risk factor for incomplete clinical success after surgery in UPA.

## Introduction

Primary aldosterone (PA), first reported in 1955, is a known cause for hypertension (1). PA is the most common form of secondary hypertension, accounting for approximately 5–15% of all hypertension cases (2). PA is divided into unilateral PA (UPA) and bilateral PA (BPA), with aldosterone-producing adenoma (APA) and bilateral adrenal hyperplasia (BAH) being the most common forms of PA. APA is mainly treated with unilateral adrenalectomy, while BAH is treated with mineralocorticoid receptor antagonist (MRA) (3). PA is associated with a greater risk of cardiovascular, cerebrovascular, renal, and metabolic disease than essential hypertension (4–8). In recent years, the reported prevalence of PA combined with cortisol co-secretion is about 10–30% in different regions (9–13). Elevated serum cortisol further increases the risk of cardiovascular disease, glucose tolerance/diabetes, and osteoporosis associated with elevated serum aldosterone (9, 14–17). Several studies have investigated certain clinical characteristics of PA combined with cortisol co-secretion. Owing to regional and demographic differences, there is no consensus on the clinical features of PA combined with cortisol co-secretion, but there seems to be an agreement on the association of larger tumor size with PA (11, 14, 18).

*KCNJ5* is the most common gene mutation in PA, and the results of a recent study in China showed that *KCNJ5* was mutated in 70.7% of APA (19). The mutation status of *KCNJ5* in PA with and without cortisol co-secretion is controversial. A study showed that APA with cortisol co-secretion had a significantly lower rate of *KCNJ5* mutations than those without (10). However, a study from Japan showed that there was no difference in *KCNJ5* mutations between APA with cortisol co-secretion and those without (20). Adrenocorticotropic hormone (ACTH) regulates both aldosterone and cortisol secretion and can further promote aldosterone secretion in combination with ACTH receptor (MC2R) (21, 22).

The cosyntropin stimulation test (CST) was first reported in 1978 (23). Currently, it is mostly used to differentiate APA or UPA from bilateral PA (24, 25). The use of CST to differentiate between the subtypes of primary hyperaldosteronism has been proposed but not fully validated and is hence currently not used as standard practice in many countries including USA.

This study was designed to further explore the clinical characteristics and postoperative outcomes of UPA with cortisol co-secretion in China and examine, for the first time, its response to CST.

## Materials and methods

### Patients

**Figure 1:**
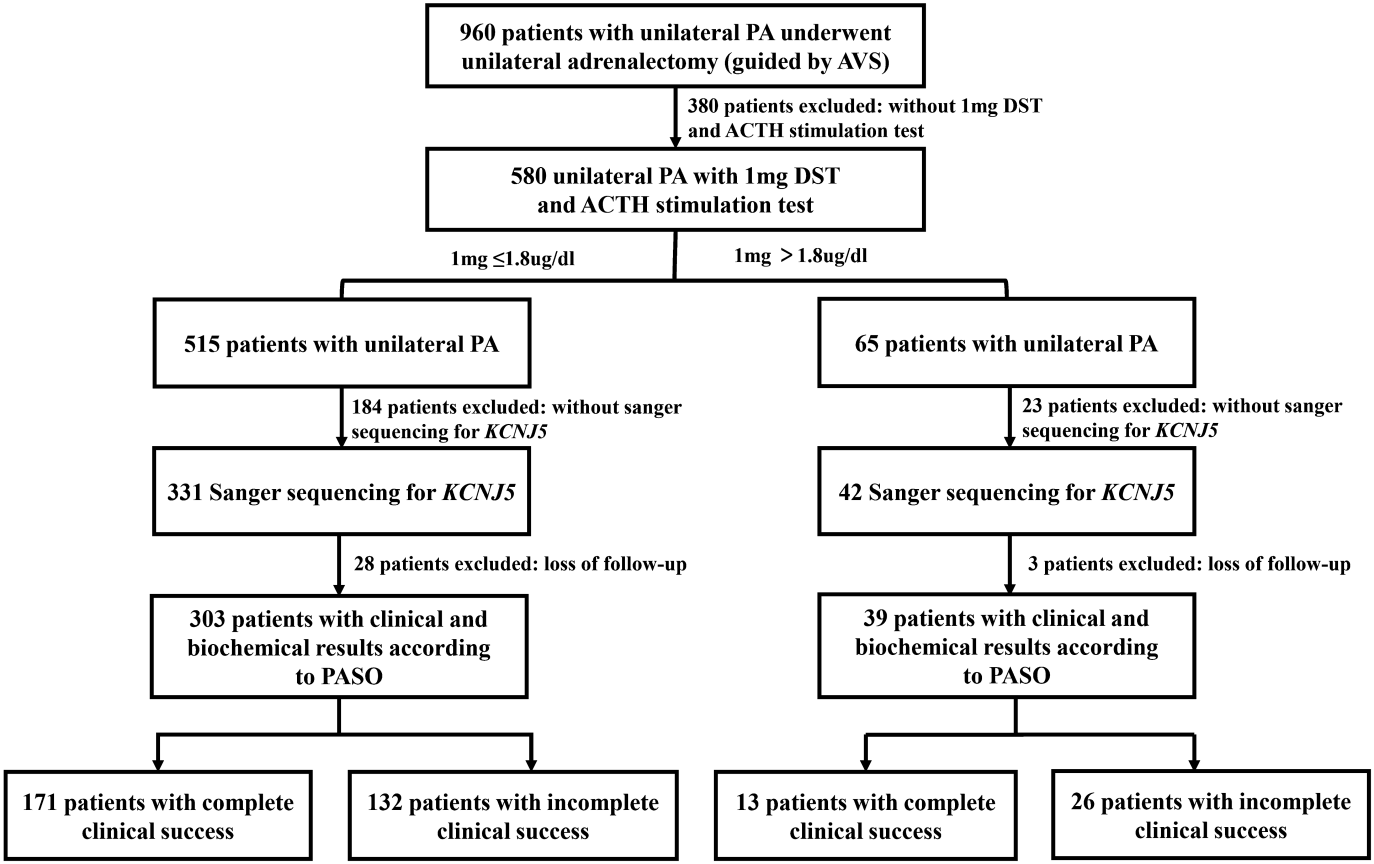
Flow chart.

We screened 960 consecutive patients with UPA who had showed lateralization on adrenal venous sampling (AVS) and underwent an adrenalectomy. Finally, we included 580 UPA patients who underwent a CST (50 IU) after 1-mg dexamethasone suppression test (DST). Of these, 373 had data of Sanger sequencing for *KCNJ5*, and 342 cases had follow-up data for at least 6 months after surgery. All patients were referred to Ruijin Hospital Affiliated to Shanghai Jiaotong University School of Medicine, from December 2010 to February 2022 (**Figure 1**). This study was approved by the Ethics Committee of Ruijin Hospital, Shanghai Jiao Tong University School of Medicine. All patients provided informed consent for participation.

### Diagnostic criteria for PA and subtype identification

PA was diagnosed according to the 2008 and 2016 endocrine society clinical practice guidelines (26, 27). The aldosterone-to-renin ratio (ARR) was used as a screening indicator for PA. Patients with ARR>30[(ng/dl)/(ng/ml/h)] were further subjected to a saline infusion test (SIT). Patients were made to lie down for at least 2 h and then, 2 L 0.9% saline was slowly infused via the peripheral vein over 4 h. PA was diagnosed if the plasma aldosterone concentration (PAC) was >10 ng/dL after infusion.

Adrenal computed tomography (CT) and AVS were used to differentiate between UPA and BPA. Cannulation was considered successful if the ratio of cortisol (adrenal vein)/cortisol (peripheral vein) was >3 without cosyntropin stimulation. Cortisol-corrected aldosterone ratio (A/C) served to correct adrenal venous aldosterone concentration for differing degrees of dilution of adrenal versus peripheral venous blood. Patients with (A/C) _adrenal vein_/(A/C) _contralateral adrenal vein_>2 were considered to have dominant secretion (26).

The primary aldosteronism surgical outcome (PASO) criteria (28) were used to assess outcomes after adrenalectomy for UPA. Complete clinical success in patients was defined normalized blood pressure and non-use of any antihypertensive medicine. Patients without hypokalemia (if present preoperatively) and normalized ARR were classified as having complete biochemical success.

### Diagnostic criteria for PA with cortisol co-secretion

The diagnosis of PA with cortisol co-secretion is based on the 2016 European Guidelines for Unexpected Adrenal Tumors (29): (1) a confirmed PA diagnosis; (2) post-dexamethasone serum cortisol levels >1.8 ug/dL; (3) absence of typical overt Cushing’s Syndrome (CS) features such as striae, skin atrophy, facial plethora, and central obesity; and (4) presence of an adrenal mass confirmed via CT before surgery, and a pathological diagnosis of the adrenal mass as an adrenal adenoma after surgery.

### ACTH stimulation test under 1-mg DST

Patients received 1 mg dexamethasone orally at midnight. The following morning at 0800 h, 4 mL (50 IU) of cosyntropin (produced by Shanghai No.1 biochemical&pharmaceutical corporation) was injected slowly via a peripheral vein within 1 min, and then peripheral venous blood samples were collected every 30 min for 2 h for cortisol, aldosterone, and dehydroepiandrosterone sulfate (DHEAS) measurements.

### Laboratory measurements

All tests were carried out in a College of American Pathologists (CAP)-accredited laboratory (CAP No. 7217913). Serum aldosterone and plasma renin activity were measured by radioimmunoassay (RIA) following the manufacturer’s instructions (Beckman Coulter). The intra-assay and inter assay coefficients of variation were 9.3 and 9.5% for aldosterone and 10.1 and 10.2% for renin activity, respectively. The respective reference values were 3.81–31.33 ng/dL and 0.1–5.5 ng/mL/h. Serum cortisol and serum ACTH were measured by immunoluminescence and radio immunoassay (RIA) following the manufacturer’s instructions (Beckman Coulter). The intra-assay and inter assay coefficients of variation were 6.7 and 7.9% for cortisol and 6.1 and 5.3% for ACTH, respectively. The respective reference values were 6.7–22.6 ug/dL and 12–78 pg/mL, respectively.

### Molecular analysis

Genetic testing of adrenal tumors was performed in specimens from UPA patients who underwent unilateral adrenalectomy. The genomic DNA was prepared using the QIAGEN DNeasy tissue kit (Qiagen, Hilden, Germany). Polymerase chain reaction was performed in a Dual 96-well GeneAmp PCR system 9700 (Applied Biosystems Courtaboeuf, France) using 20 ng of template DNA from each frozen sample per reaction. The products were sequenced on a 3730xl DNA analyzer (Applied Biosystems). All of the sequences were analyzed using sequencing analysis software version 5.2 (Applied Biosystems). The tumor samples were screened for mutations in the hotspot area of *KCNJ5*. The specific primer sequences are listed as follows:

KCNJ5-F: GGTGACCTGGACCATGTTGGCG

KCNJ5-R: CTTGGCAGGTCATGCCTGTGGC

### Statistical analysis

Patients were categorized separately based on whether their serum cortisol level was >1.8 µg/dL after the 1-mg DST and whether they had complete clinical success after surgery. SPSS software ((version 26.0; IBM Corporation, Armonk, NY, USA) was used for statistical analyses. Normally distributed data are presented as means ± standard deviation (SD) and non-normally distributed data are expressed as medians (interquartile range: 25^th^–75^th^ percentile). Categorical variables are presented as frequencies or percentages. The *t*-test and chi-square test were used for comparisons between two groups for continuous and categorical variables, respectively. The diagnostic value of PAC after ACTH stimulation for cortisol co-secretion in UPA was assessed based on receiver operating characteristic (ROC) curves and the area under the ROC curve (AUC). Multivariable regression analysis (method: LR) was performed to investigate the factors influencing serum cortisol >1.8 ug/dL after 1-mg DST and complete clinical success after surgery. The ROC curves and line plots were plotted using MedCalc (15.2) and GraphPad (8.0), respectively. A *P* value of <0.05 was considered to indicate statistically significant differences.

## Results

### Baseline characteristics and demographics

Our study included a total of 580 patients diagnosed with UPA. Based on the serum cortisol level after 1-mg DST, there were 65 (11.2%) UPA cases with 1-mg DST>1.8 ug/dL and 515 (88.8%) UPA cases without 1-mg DST>1.8 ug/dL. The baseline characteristics between the two groups are presented in **Table 1**. Patients in the UPA with 1-mg DST>1.8 ug/dL group were older (52.8 ± 8.7 vs. 46.7 ± 11.2 years, P<0.001), had longer duration of hypertension (10.0 [4.5–15.5] vs. 6.0 [2.0–10.0] years, P<0.05) and a higher prevalence of diabetes mellitus (18.5% vs. 9.1%, P<0.05) than the UPA without 1-mg DST>1.8 ug/dL group. The UPA with 1-mg DST>1.8 ug/dL group had higher PAC (55.0 [34.6–90.6] vs. 43.4 [30.0–69.0] ng/dL, P<0.05); midnight cortisol (3.2 [2.3–4.9] vs. 2.1 [1.4–3.6] ug/dL, P<0.001); and serum cortisol after 1-mg DST (2.2 [2.0–3.3] vs. 1.0 [0.8–1.2] ug/dL, P<0.001). While, ACTH (27.2 ± 12.3 vs. 35.2 ± 22.7 pg/mL, P<0.05) and DHEAS 128.2 [74.6–190.4] vs. 182.6 [124.7–258.1] ug/dL, P<0.001] were lower in the UPA with 1-mg DST>1.8 ug/dL. However, there was no difference between the two groups with respect to systolic blood pressure (SBP), diastolic blood pressure (DBP), serum sodium, serum potassium, plasma renin activity (PRA), ARR, serum cortisol (0800 h and 1600 h) and 24-h urinary free cortisol (24h-UFC). The maximum diameter of the adrenal tumor was larger in UPA with 1-mg DST>1.8 ug/dL (1.7 [1.3–2.2] vs. 1.4 [1.1–1.7] cm, P<0.001).

**Table 1:** Baseline characteristics of UPA with different serum cortisol levels after the 1-mg DST.

Characteristics	UPA without 1-mg DST>1.8	UPA with 1-mg DST>1.8	*P*
Case number, *N* (%)	515 (88.8%)	65 (11.2%)	
Age (year)	46.7 ± 11.2	52.8 ± 8.7	<0.001
Male, *N* (%)	282 (54.8%)	32 (49.2%)	0.399
BMI (kg/m^2^)	24.5 ± 3.7	24.0 ± 2.9	0.194
Duration of hypertension (year)	6.0 (2.0-10.0)	10.0 (4.5-15.5)	0.002
History of diabetes mellitus, *N* (%)	47 (9.1%)	12 (18.5%)	0.019
SBP (mmHg)	172.6 ± 22.6	174.2 ± 21.8	0.585
DBP (mmHg)	105.7 ± 15.8	104.7 ± 13.4	0.627
PAC (ng/dL)	43.4 (30.0-69.0)	55.0 (34.6-90.6)	0.016
PRA (ng/mL/h)	0.24 (0.08-0.59)	0.21 (0.10-0.48)	0.561
ARR [(ng/ml)/(ng/ml/h)]	192.3 (74.2-605.5)	241.7 (84.6-906.2)	0.213
Serum cortisol 0800 h (ug/dL)	11.5 (8.7-14.3)	11.6 (9.2-15.0)	0.490
Serum cortisol 1600 h (ug/dL)	5.5 (4.3-7.3)	6.0 (4.5-8.0)	0.192
Serum cortisol midnight (ug/dL)	2.1 (1.4-3.6)	3.2 (2.3-4.9)	<0.001
1mg DST (ug/dL)	1.0 (0.8-1.2)	2.2 (2.0-3.3)	<0.001
ACTH (pg/mL)	35.2 ± 22.7	27.2 ± 12.3	0.006
24h-UFC (ug/24 h)	75.3 (57.0-96.6)	77.7 (56.9-94.7)	0.933
DHEAS (ug/dL)	182.6 (124.7-258.1)	128.2 (74.6-190.4)	<0.001
Serum sodium (mmol/L)	143.3 ± 2.8	143.0 ± 3.3	0.479
Serum potassium (mmol/L)	3.0 ± 0.4	3.0 ± 0.4	0.774
Tumor size (cm)	1.4 (1.1-1.7)	1.7 (1.3-2.2)	<0.001

BMI, body mass index; SBP, systolic blood pressure; DBP, diastolic blood pressure; PAC, plasma aldosterone concentration; PRA, plasma renin activity; ARR, aldosterone renin ratio; 1-mg DST, 1-mg dexamethasone suppression test; ACTH, adrenal corticotropic hormone; 24h-UFC, 24-h urinary free cortisol; DHEAS, dehydroepiandrosterone sulfate.

### Response of UPA to CST at different serum cortisol levels

The patient’s AVS parameters are shown in **Supplementary Table S1**. There was no difference in aldosterone, cortisol, and A/C between the dominant and nondominant adrenal veins in the two groups. There was no difference in selection index (SI) on the dominant side, but it was lower on the nondominant side. Furthermore, there was no difference in lateralization index (LI) and contralateral suppression index (CSI) in the two groups.

**Figure 2:**
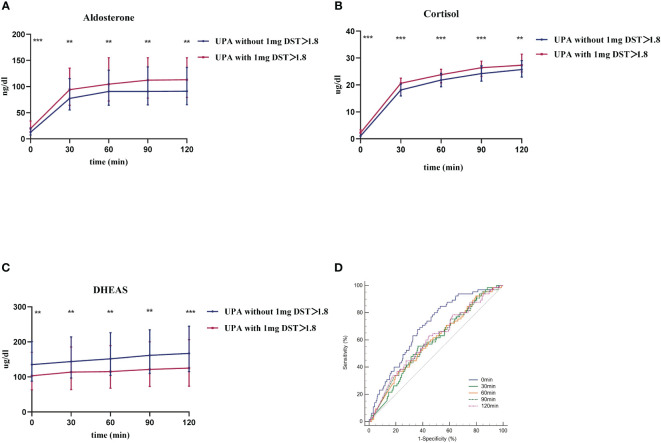
The response of UPA to cosyntropin stimulation test at different cortisol levels. **(A–C)** respective represent aldosterone, cortisol, and DHEAS of both groups after cosyntropin stimulation test under the influence of 1-mg dexamethasone suppression test. Data are shown as median with quartile range. **(D)** represents the ROC curve of aldosterone level at 0 min, 30 min, 60 min, 90 min, and 120 min after cosyntropin injection in the differential diagnosis between UPA with 1-mg DST>1.8 and those without. **P<0.05. ***P<0.001.

The changes in aldosterone, cortisol, and DHEAS levels after CST between the two groups are shown in **Figure 2**. Post cosyntropin administration, aldosterone, and cortisol were higher and DHEAS was lower in the UPA with 1-mg DST>1.8 ug/dL group (**Figures 2A, B**) than the UPA without 1-mg DST>1.8 group (**Figure 2C**) at each time point (P<0.05).

We first evaluated the diagnostic value of CST in distinguishing UPA with cortisol co-secretion and those without. **Figure 2D** shows the ROC curve of PAC at each point after cosyntropin injection for the differential diagnosis of UPA with or without cortisol co-secretion. The AUCs (95% confidence interval [CI]) of aldosterone at 0 min, 30 min, 60 min, 90 min, and 120 min were 0.696 (0.657–0.733), 0.587 (0.546–0.628), 0.591 (0.550–0.632), 0.594 (0.553–0.634), and 0.603 (0.562–0.643), respectively. Among these curves, aldosterone at 0 min was the best marker for the diagnosis of UPA with cortisol co-secretion. The optimal cut-off was 11.99 ng/dL, which displayed a sensitivity of 83.08% and specificity of 48.54%.

### Comparison of preoperative and postoperative parameters for UPA

We analyzed the postoperative parameters in 342 UPA with *KCNJ5* gene sequencing (**Table 2**), and a comparison of their baseline characteristics are presented in **Supplementary Table S2**. After surgery, SBP, DBP, PAC, and ARR were significantly decreased and PRA was elevated in both groups (P<0.001). Postoperative ACTH increased significantly in both groups (P<0.001), and DHEAS did not change significantly, but DHEAS remained lower in the UPA with 1-mg DST>1.8 ug/dL group. After surgery, serum sodium was significantly decreased and serum potassium was obviously increased in both groups. Complete clinical success rate was higher in the group of UPA without 1-mg DST>1.8 ug/dL after surgery (56.4% vs. 33.3%, P<0.05), but there was no difference in complete biochemical success between the two groups. The total mutation rate of *KCNJ5* was 71.6% (245/342), but there was no significant difference between the two groups (71.3% vs. 74.4%, P>0.05).

**Table 2:** Comparison of post-operative parameters between the two groups.

	UPA without 1-mg DST>1.8 (N=303)			UPA with 1-mg DST>1.8 (N=39)			
	Baseline	Follow-up	*P^1^*	Baseline	Follow-up	*P^1^*	*P* ^2^
SBP (mmHg)	171.5 ± 22.7	130.0 ± 15.0	<0.001	170.0 ± 20.4	136.0 ± 16.7	<0.001	0.022
DBP (mmHg)	105.5 ± 14.6	82.4 ± 9.8	<0.001	104.7 ± 13.7	83.0 ± 10.3	<0.001	0.726
PAC (ng/dL)	42.1 (29.8-68.3)	8.9 (5.3-12.6)	<0.001	54.5 (33.5-95.6)	9.6 (5.3-12.2)	<0.001	0.913
PRA (mg/dL/h)	0.3 (0.1-0.6)	1.8 (0.9-3.0)	<0.001	0.2 (0.1-0.5)	1.8 (1.0-2.9)	<0.001	0.975
ARR [(ng/dL)/(mg/dL/h)]	173.5 (68.8-521.7)	5.7 (3.2-10.5)	<0.001	207.4 (82.1-705.9)	5.2 (3.8-11.2)	<0.001	0.917
Serum cortisol 0800 h (ug/dL)	11.3 (8.7-14.2)	11.7 (9.4-13.9)	0.313	12.7 (9.8-16.0)	11.8 (9.5-14.6)	0.617	0.645
Serum cortisol 1600 h (ug/dL)	5.5 (4.3-7.2)	6.3 (5.0-8.0)	0.005	6.4 (4.5-8.5)	6.6 (4.8-8.3)	0.880	0.956
Serum cortisol midnight (ug/dL)	2.1 (1.4-3.5)	2.2 (1.3-3.7)	0.572	3.1 (2.3-4.9)	2.3 (1.6-5.6)	0.099	0.251
ACTH (pg/mL)	33.7 ± 17.9	52.0 ± 29.3	<0.001	27.6 ± 11.4	44.5 ± 19.4	<0.001	0.184
DHEAS (ug/dL)	179.6 (126.5-256.4)	178.2 (114.4-243.9)	0.314	137.0 (77.6-207.4)	121.5 (78.9-186.8)	0.617	0.040
Serum sodium (mmol/L)	143.3 ± 3.0	140.5 ± 2.1	<0.001	143.0 ± 3.3	140.0 ± 1.9	<0.001	0.318
Serum potassium (mmol/L)	3.0 ± 0.4	4.2 ± 0.4	<0.001	3.0 ± 0.3	4.2 ± 0.4	<0.001	0.536
Complete clinical success, *N* (%)		171/303 (56.4%)			13/39 (33.3%)		0.006
Complete biochemistry success, *N* (%)		296/303 (97.7%)			38/39 (97.4%)		1.000
*KCNJ5*, *N* (%)		216/303 (71.3%)			29/39 (74.4%)		0.689

^1^Comparison between baseline and follow up data within each group^2^. Comparison of follow-up information between the two groups. SBP, systolic blood pressure; DBP, diastolic blood pressure; PAC, plasma aldosterone concentration; PRA, plasma renin activity; ARR, aldosterone renin ratio; ACTH, adrenal corticotropic hormone; DHEAS, dehydroepiandrosterone sulfate.

### Factors influencing UPA with 1-mg DST>1.8 ug/dL

**Figure 3:**
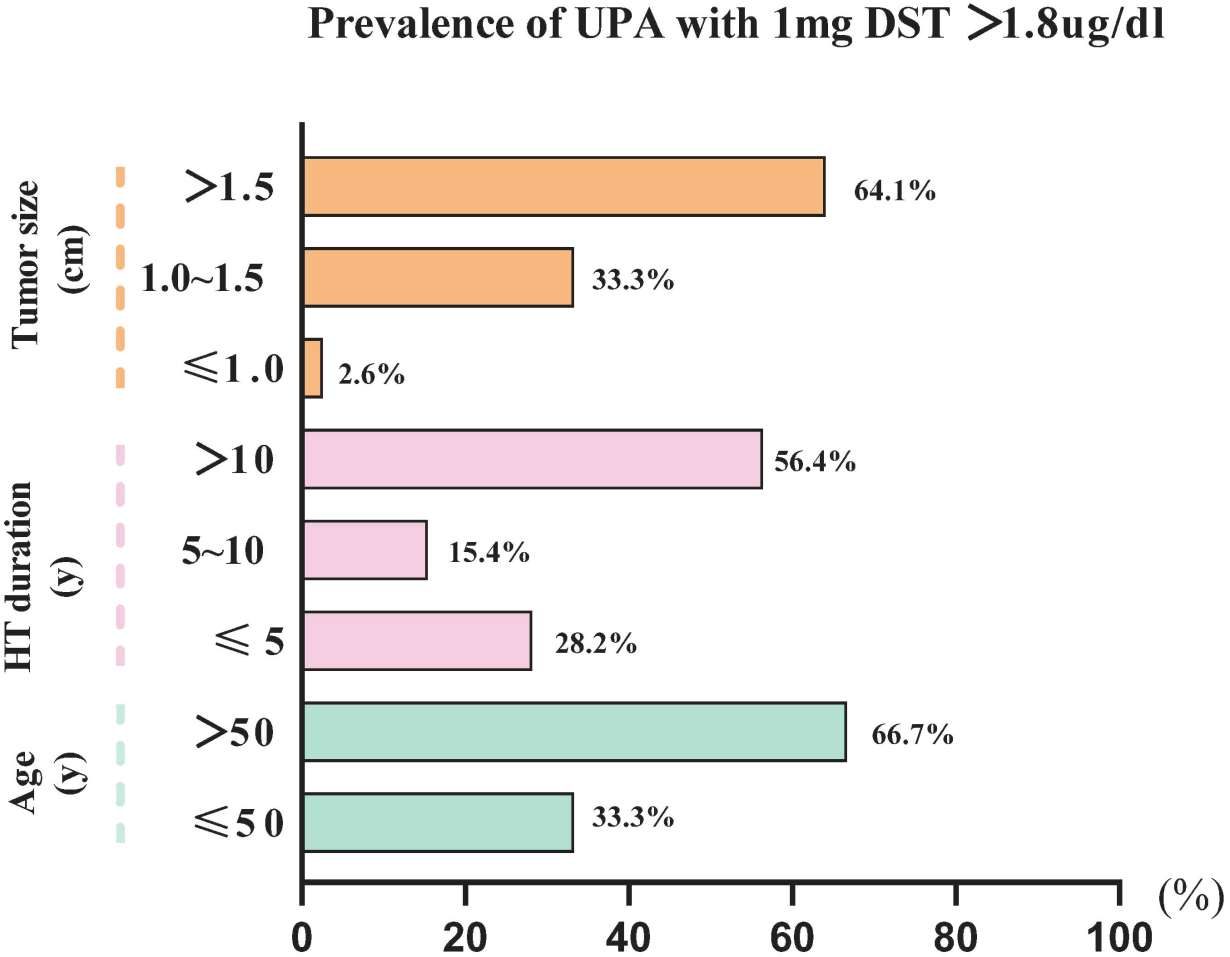
The characteristics of UPA with 1-mg DST>1.8 ug/dL. Data are expressed as a percentage.

We performed a binary logistic regression analysis of the factors associated with 1-mg DST>1.8 ug/dL (**Table 3**). Univariate regression analysis revealed that age (1.075 [1.036–1.115], P<0.001); duration of hypertension (1.069 [1.025–1.115], P<0.05); tumor size (3.921 [2.180–7.054], P<0.001); ACTH (0.971 [0.945–0.998], P<0.05); and DHEAS (0.995 [0.991–0.999], P<0.05) were significantly associated with UPA with 1-mg DST>1.8 ug/dL. Further multiple regression analysis revealed that age (1.094 [1.049–1.141], P<0.001) and tumor size (4.508 [2.370–8.576], P<0.001) were independent risk factors for 1-mg DST>1.8 ug/dL, and ACTH (0.967 [0.938–0.997], P<0.05) was a protective factor for it. Our further analysis of age, tumor sizes, and duration of hypertension revealed that the UPA with 1-mg DST>1.8 ug/dL group was characterized by the following clinical features (**Figure 3**): age>50 years (66.7%), duration of hypertension>10 years (56.4%), and maximum tumor diameter>1.5 cm (64.1%).

**Table 3:** Factors associated with 1-mg DST>1.8 ug/dL in 342 UPA.

	Univariate		Multivariate	
	Odds Ratio(95% CI)	*P*	Odds Ratio(95% CI)	*P*
Age (year)	1.075 (1.036-1.115)	<0.001	1.094 (1.049-1.141)	<0.001
Male (yes)	0.998 (0.511-1.948)	0.995		
BMI (kg/m^2^)	0.958 (0.870-1.055)	0.381		
Duration of hypertension (year)	1.069 (1.025-1.115)	0.002		
PAC (ng/dL)	1.000 (1.000-1.001)	0.155		
PRA (mg/dL/h)	1.092 (0.646-1.846)	0.742		
Serum cortisol 0800 h (ug/dL)	1.062 (0.982-1.148)	0.131		
Serum cortisol 1600 h (ug/dL)	1.099 (0.984-1.228)	0.094		
Serum cortisol midnight (ug/dL)	1.056 (0.979-1.138)	0.158		
ACTH (pg/mL)	0.971 (0.945-0.998)	0.036	0.967 (0.938-0.997)	0.032
Serum potassium (mmol/L)	0.682 (0.285-1.632)	0.390		
Serum sodium (mmol/L)	0.961 (0.855-1.079)	0.499		
DHEAS (ug/dL)	0.995 (0.991-0.999)	0.020		
Tumor size (cm)	3.921 (2.180-7.054)	<0.001	4.508 (2.370-8.576)	<0.001
*KCNJ5* mutation, yes	1.463 (0.688-3.110)	0.323		

BMI, body mass index; PAC, plasma aldosterone concentration; PRA, plasma renin activity; ACTH, adrenal corticotropic hormone; DHEAS, dehydroepiandrosterone sulfate.

### Factors influencing complete clinical success of UPA after surgery

We analyzed the factors associated with complete clinical success after surgery in UPA (**Table 4**). Univariate logistic regression analysis revealed that age (1.060 [1.037–1.083], P<0.001); male sex (3.591 [2.287–5.639], P<0.001); BMI (1.249 [1.160–1.344], P<0.001); duration of hypertension (1.120 [1.079–1.163], P<0.001); renin activity (1.712 [1.141–2.569], P<0.05); 1-mg DST>1.8 ug/dL (2.591 [1.282–5.235], P<0.05); and *KCNJ5* mutation (0.342 [0.209–0.558], P<0.001) were significantly association with complete clinical success. Tumor size was not associated with complete clinical success (P>0.05). Multiple regression analysis showed that male sex, higher BMI, longer duration of hypertension, cortisol co-secretion (1-mg DST>1.8 µg/dL), and absence of *KCNJ5* mutation were independent predictors for complete clinical success.

**Table 4:** Factors associated with complete clinical success in 342 UPA.

	Univariate		Multivariate	
	Odds Ratio(95% CI)	*P*	Odds Ratio(95% CI)	*P*
Age (year)	1.060 (1.037-1.083)	<0.001		
Male, yes	3.591 (2.287-5.639)	<0.001	2.496 (1.434-4.355)	0.001
BMI (kg/m^2^)	1.249 (1.160-1.344)	<0.001	1.169 (1.076-1.271)	<0.001
Duration of hypertension (year)	1.120 (1.079-1.163)	<0.001	1.116 (1.070-1.164)	<0.001
PAC (ng/dL)	1.000 (1.000-1.001)	0.601		
PRA (mg/dL/h)	1.712 (1.141-2.569)	0.009		
Serum cortisol 0800 h (ug/dL)	1.036 (0.982-1.092)	0.195		
ACTH (pg/mL)	1.003 (0.991-1.015)	0.666		
1-mg DST>1.8, yes	2.591 (1.282-5.235)	0.008	2.461 (1.094-5.535)	0.029
Tumor size (cm)	0.854 (0.564-1.294)	0.457		
*KCNJ5* mutation, yes	0.342 (0.209-0.558)	<0.001	0.516 (0.294-0.906)	0.021
DHEAS (ug/dL)	1.000 (0.998-1.002)	0.977		
Serum potassium (mmol/L)	1.066 (0.630-1.803)	0.811		
Serum sodium (mmol/L)	0.992 (0.924-1.065)	0.992		

BMI, body mass index; PAC, plasma aldosterone concentration; PRA, plasma renin activity; ACTH, adrenal corticotropic hormone; 1mg DST, 1-mg dexamethasone suppression test;

DHEAS, dehydroepiandrosterone sulfate.

## Discussion

An epidemiological survey showed that the prevalence of PA in patients with newly diagnosed hypertension in China was at least 4% (30). According to European guidelines (29), we chose 1.8 as the cortisol cut-off point after 1-mg DST and divided the patients into two groups. In this study, the prevalence of UPA with cortisol co-secretion was 11.20%, which is similar to reports from Greece (31), but lower than that in Japan (12, 18) and Germany (17). In a study with 82 PA subjects in Taiwan, the prevalence of APA with 1-mg DST>1.8 ug/dL was 26.8% (10). Differences in prevalence may be related to ethnicity and geography. Our study suggests that while the prevalence of UPA with cortisol co-secretion is not low in China, this particular subtype of PA requires further research attention.

Our study found that UPA patients with cortisol co-secretion were older, had a longer duration of hypertension, and a higher prevalence of diabetes; these characteristics were similar to some of the previous findings (9, 11). In our study, UPA with cortisol co-secretion had a higher PAC, while PRA and ARR did not show significant differences with UPA without cortisol co-secretion. A study by Tsai et al. (9) also suggested that PA with cortisol co-secretion had higher aldosterone concentrations, while the results from O’Toole (13) and Peng (10) did not support this view. Taken together, whether PA with cortisol co-secretion is accompanied by higher aldosterone levels remains a controversial issue. Serum DHEAS concentration was lower in the UPA with cortisol co-secretion group, which was associated with high serum cortisol concentrations suppressing ACTH in our group. Our study found that UPA with cortisol co-secretion had larger adrenal tumors than those without cortisol co-secretion in China. Some studies have come to a similar conclusion. Tang et al. (14) found that the tumor sizes of APA with cortisol co-secretion were 6 mm larger than those of pure APA (24.50 ± 11.34 vs. 18.92 ± 7.98 mm, P<0.05). This was also consistent with Yasuda et al’s study (11), in that PA patients with cortisol co-secretion had a larger tumor than those without cortisol co-secretion (2.7 ± 0.1 vs. 1.4 ± 0.1 cm, P<0.05).

Aldosterone is produced by the adrenal zona glomerulosa, which is regulated by ACTH, serum potassium, and angiotensin II (32). We found that after cosyntropin was administered per peripheral IV, aldosterone and cortisol rose more significantly in UPA with cortisol co-secretion, while DHEAS levels were lower in this group. This suggests that ACTH promotes the production of both aldosterone and cortisol from the adrenocortical globus and fasciculus, and that this effect was more pronounced in patients with UPA with cortisol co-secretion. A study by St. Jean et al. (33) suggested that ACTH only promotes the production of aldosterone in PA without cortisol co-secretion, while it promotes both aldosterone and cortisol secretion in PA with cortisol co-secretion. We speculated that PA patients with cortisol co-secretion have a higher number of ACTH receptors or a higher sensitivity of ACTH receptors than those with PA without cortisol co-secretion, which is why the former can produce more aldosterone and cortisol. In addition, we believe this is the first study to discuss whether CST can be used to distinguish between UPA with and without cortisol co-secretion. Our results showed that CST was not an effective differentiating tool in this regard. Although our study did not find an association between *KCNJ5* and cortisol co-secretion, some studies have found an association. Inoue et al. (34) found that *KCNJ5*-muted-APA had lower aldosterone concentration than *KCNJ5*-wild-APA after dexamethasone, suggesting that *KCNJ5*-muted-APA is more responsive to endogenous ACTH and that the ACTH pathway may be more sensitive and easily activated. The cause of PA with cortisol co-secretion is controversial. Our results revealed that age, tumor size, and ACTH were significantly correlated with PA with cortisol co-secretion. This is also similar to the study of Peng et al. (10), who concluded that tumor size was positively correlated with PA with cortisol co-secretion.

In our study, UPA patients with cortisol co-secretion had a lower complete clinical success rate than those without cortisol co-secretion after surgery. The results were similar to the findings of a Taiwanese study (10), although its findings were not significant (62.5% vs. 38.5%, P>0.05). This study reveals that the duration of hypertension and cortisol co-secretion were independent risk factors for complete clinical remission in UPA after surgery, while *KCNJ5* mutation was a protective factor. This finding is consistent with the findings of Peng et al. (10) However, Peng et al. also revealed that the *KCNJ5* mutation was negatively associated with cortisol co-secretion (OR=0.23, 95%CI: 0.06–0.83, P=0.024). This could be because their sample size was smaller than ours. In addition, the total mutation rate of *KCNJ5* (71.6%) in this study was consistent with previous findings from our center (19), and UPA with and without cortisol co-secretion had similar *KCNJ5* mutation rates, which is consistent with a study from Japan (20).

### Limitations

First, we did not discuss the characteristics of immunohistochemistry (CYP11B1 and CYP11B2) in UPA with cortisol co-secretion. Second, some relevant genetic mutations such as *ATP1A1*, *ATP2B3*, *CTNNB1*, *CACNA1D*, and *PRKACA* were not studied. Third, our study was limited to the clinical features and prognosis of UPA with cortisol co-secretion, and did not explore the underlying mechanisms. Last, the subjects in our study did not undergo the 1-mg DST and AST after surgery. Therefore, more studies are needed to explore and summarize the characteristics and mechanisms of PA with cortisol co-secretion.

## Conclusions

In China, there is a high prevalence of UPA with cortisol co-secretion, and their clinical features are distinctly different from those without cortisol co-secretion. UPA patients with cortisol co-secretion are more responsive to ACTH than those without. Cortisol co-secretion is associated with a decreased chance of complete clinical success in UPA after surgery. The 1-mg DST should be routinely performed in the daily practice of PA to detect cortisol co-secretion early.

## Data availability statement

The raw data supporting the conclusions of this article will be made available by the authors, without undue reservation.

## Ethics statement

The studies involving humans were approved by Ethics Committee of Ruijin Hospital, Shanghai Jiao Tong University School of Medicine. The studies were conducted in accordance with the local legislation and institutional requirements. The participants provided their written informed consent to participate in this study.

## Author contributions

YJ: Writing – original draft, Writing – review & editing. LZ: Data curation, Formal analysis, Funding acquisition, Methodology, Software, Writing – original draft, Writing – review & editing. CZ: Methodology, Project administration, Resources, Software, Writing – review & editing. TS: Conceptualization, Data curation, Formal analysis, Writing – review & editing. LJ: Data curation, Formal analysis, Investigation, Writing – review & editing. WZ: Resources, Software, Writing – review & editing. XZ: Resources, Software, Supervision, Writing – review & editing. LW: Formal analysis, Resources, Software, Writing – review & editing. WW: Conceptualization, Data curation, Formal analysis, Funding acquisition, Investigation, Methodology, Project administration, Resources, Software, Supervision, Validation, Visualization, Writing – original draft, Writing – review & editing.
