# Supplementary material for: The influence of cortisol co-secretion on clinical characteristics and postoperative outcomes in unilateral primary aldosteronism
Source: Front Endocrinol (Lausanne). 2024 Apr 30;15:1369582. doi: 10.3389/fendo.2024.1369582 (PMC11091262; doi:10.3389/fendo.2024.1369582)
Supplement: Supplementary file 1 [file Table_1.docx]

Supplementary

| **Table S1**. Adrenal venous parameters and interpretation according to cortisol status in AVS | | | |
| --- | --- | --- | --- |
|  | UPA without 1-mg DST>1.8  (N=515) | UPA with 1-mg DST>1.8  (N=65) | *P* |
| Lateralization (Left/Right) | 293/222 | 34/31 | 0.482 |
| **Dominant adrenal vein** |  |  |  |
| Aldosterone (ng/dL) | 4545.96 (2005.40, 10767.68) | 4825.68 (2074.53, 9391.06) | 0.742 |
| Cortisol (µg/dL) | 357.04 (167.31, 581.88) | 306.67 (129.99, 584.75) | 0.375 |
| A/C | 14.20 (5.90, 34.23) | 14.27 (6.96, 31.81) | 0.606 |
| **Non-dominant adrenal vein** |  |  |  |
| Aldosterone (ng/dL) | 358.36 (141.00,583.24) | 278.08 (116.95, 570.06) | 0.437 |
| Cortisol (µg/dL) | 367.24 (178.56, 655.94) | 316.98 (101.47, 536.60) | 0.050 |
| A/C | 0.91 (0.62, 1.38) | 1.02 (0.68, 2.00) | 0.068 |
| **Inferior vena cava (IVC)** |  |  |  |
| Aldosterone (ng/dL) | 42.01 (28.23, 63.58) | 45.44 (32.01, 62.05) | 0.363 |
| Cortisol (µg/dL) | 12.13 (9.08, 14.84) | 12.48 (9.66, 15.03) | 0.634 |
| A/C | 3.61 (2.44, 5.66) | 3.92 (2.74, 5.86) | 0.450 |
| **Selectivity index** |  |  |  |
| Dominant AV | 30.28 (16.71, 43.73) | 26.35 (14.35, 37.54) | 0.145 |
| Nondominant AV | 31.04 (18.51, 49.31) | 23.84 (9.13, 41.07) | 0.008 |
| **LI** | 15.02 (5.99, 35.03) | 12.55 (6.57, 30.17) | 0.596 |
| **CSI** | 0.26 (0.16, 0.45) | 0.27 (0.20, 0.47) | 0.229 |

A/C, aldosterone/cortisol; AV, adrenal vein; LI, lateralization index; CSI, contralateral suppression index.

**Table S2.** Comparison of baseline information between the two groups of 342 UPA patients.

|  | UPA without 1-mg DST>1.8 | UPA with 1-mg DST>1.8 | *P* |
| --- | --- | --- | --- |
| Case number, *N* (%) | 303 (88.6%) | 39 (11.4%) |  |
| Age (year) | 45.5±10.9 | 53.1±9.4 | ＜0.001 |
| Male, *N* (%) | 163 (53.8%) | 21 (53.8%) | 0.995 |
| BMI (kg/m^2^) | 24.5±3.8 | 24.0±2.8 | 0.383 |
| Duration of hypertension (year) | 6.0 (2.0-10.0) | 10.0 (3.0-17.0) | 0.015 |
| History of diabetes mellitus, *N* (%) | 26 (8.6%) | 9 (23.1%) | 0.010 |
| SBP (mmHg) | 171.5±22.7 | 170.0±20.4 | 0.648 |
| DBP (mmHg) | 105.5±14.6 | 104.7±13.7 | 0.724 |
| PAC (ng/dL) | 420.9 (298.1-683.1) | 564.7 (334.9-955.6) | 0.081 |
| PRA (ng/mL/h) | 0.26 (0.09-0.59) | 0.24 (0.11-0.49) | 0.951 |
| ARR [(ng/dL)/(mg/dL/h)] | 1734.9 (687.9-5217.0) | 2073.6 (820.9-7058.8) | 0.737 |
| Serum cortisol 0800 h (ug/dL) | 11.3 (8.7-14.2) | 12.7 (9.8-16.0) | 0.094 |
| Serum cortisol 1600 h (ug/dL) | 5.5 (4.3-7.2) | 6.4 (4.5-8.5) | 0.078 |
| Serum cortisol 0000 h (ug/dL) | 2.1 (1.4-3.5) | 3.1 (2.3-7.9) | ＜0.001 |
| 1mg DST (ug/dL) | 1.0 (0.7-1.2) | 2.2 (2.0-3.3) | ＜0.001 |
| ACTH (pg/mL) | 33.7±17.9 | 27.6±11.4 | 0.038 |
| 24h-UFC (ug/24 h) | 76.0 (55.0-96.8) | 84.0 (59.1-102.3) | 0.350 |
| DHEAS (ug/dL) | 179.6 (126.5-256.4) | 137.0 (77.6-207.4) | 0.003 |
| Serum sodium (mmol/L) | 143.3±3.0 | 142.9±3.3 | 0.503 |
| Serum potassium (mmol/L) | 3.0±0.4 | 3.0±0.3 | 0.391 |
| Tumor size (cm) | 1.5±0.5 | 1.9±0.7 | 0.001 |

BMI, body mass index; SBP, systolic blood pressure; DBP, diastolic blood pressure; PAC, plasma aldosterone concentration; PRA, plasma renin activity; ARR, aldosterone renin ratio; 1mg DST, 1mg dexamethasone suppression test; ACTH, adrenal corticotropic hormone; 24h-UFC, 24-h urinary free cortisol; DHEAS, dehydroepiandrosterone sulfate.
